# Evaluation of a population-level strategy to promote tobacco treatment use among insured smokers: a pragmatic, randomized trial

**DOI:** 10.1186/s12889-018-5119-0

**Published:** 2018-02-08

**Authors:** Jennifer B. McClure, Melissa L. Anderson

**Affiliations:** Kaiser Permanente Washington Health Research Institute (formerly, Group Health Research Institute), 1730 Minor Ave., Suite 1600, Seattle, WA 98101 USA

**Keywords:** Smoking cessation, Smoking, Health insurance, Motivation, Brief intervention

## Abstract

**Background:**

Most smokers do not use evidence-based smoking cessation treatment. Increasing utilization of these services is an important public health goal. Health care systems and insurers are well positioned to support this goal within their patient populations. We tested whether a brief, mail-based intervention increased utilization of tobacco cessation services among insured smokers.

**Methods:**

Adult smokers were identified via automated health plan data and randomized to one of five treatment arms (*n* = 4767). Randomization was stratified by gender, age, and type of health plan coverage. Three arms received a letter containing motivational content and treatment referral information. Motivational content emphasized either the financial, health, or values-based benefits of quitting. One arm received a referral letter with no motivational content, and one arm received no letter. Enrollment in the referred tobacco cessation program was monitored for 5 months. Treatment was available to all participants through their insurance.

**Results:**

Across all four letter conditions, 0.8% of participants enrolled in tobacco treatment compared to 0.9% in the no letter reference group (*p* = .69). No single letter condition was superior to the others (*p* = .71), but treatment uptake was greater among participants who received their care and coverage from the health plan versus those with insurance coverage only (1.2% vs. 0.3%, *p* < .01).

**Conclusions:**

A one-time, mailed letter is not a cost-effective strategy for promoting use of covered smoking cessation treatment within large health plan populations, particularly when the message source is an insurance provider only and does not also provide clinical care. Health plans and insurers should consider alternative outreach efforts to promote treatment uptake among smokers.

**Trial registrations:**

TRN registered retrospectively with ISRCTN registry (www.isrctn.com). Registered on 11/01/2018. Registration number: ISRCTN32311137.

## Background

Nearly 17% of adults smoke [1] and tobacco use remains the leading preventable cause of death and illness in the U.S. [2]. Smoking kills an estimated 480,000 Americans annually and results in more than $300 billion in direct health care costs and lost productivity each year [3]. Treatment for nicotine dependence is cost-effective [4] and is recommended for all smokers, [5] more than half of whom make an attempt to quit smoking each year [6]. Unfortunately, most smokers fail to utilize evidence-based treatment when trying to quit. Less than one third use a medication approved by the Food and Drug Administration and less than 10% use behavioral treatment [7]. Best practice and clinical guidelines dictate smokers should be offered a combination of these interventions, [5] but less than 6% of smokers utilize this comprehensive care [7]. Thus, improving uptake of evidence-based smoking cessation treatment is an important public health goal. Health care systems and health insurers are particularly well positioned to support this goal within the patient populations they serve. As such, it is important to evaluate population-level interventions to promote treatment utilization which can be broadly disseminated within these organizations.

Prior research showed that smoking cessation treatment use varies based on the extent of one’s insurance coverage, with the highest rates of utilization (10%) observed among those provided full coverage for comprehensive counseling and medication and the lowest rates (2.4%) observed among those with only partial insurance coverage [8]. However, this study was conducted nearly twenty years ago, before full coverage was widely available. Insurance coverage may not have the same impact on treatment uptake now that tobacco cessation treatment is mandated as a no-cost preventive care service in the United States under the Affordable Care Act. As evidence of this, an estimated 4% of smokers in our health plan have enrolled in the covered comprehensive tobacco treatment program in recent years. We do not know how this rate compares to utilization within other health care systems since those data are not public, but based on the population-level estimates presented above, it is clear that most smokers do not utilize evidence-based comprehensive tobacco cessation services when making a quit attempt. This is consistent with the fact that most smokers prefer to try to quit on their own, without the aid of treatment [9], but unaided quit attempts are less successful [5]. Thus, more needs to be done to encourage use of these best practice treatment programs. Moreover, interventions are needed which are not only effective, but are also relatively low cost and standardized--characteristics which will facilitate replication and wide-spread adoption across settings if the interventions are effective.

Recent studies show brief interventions can increase smoking cessation uptake, [10–12] but these trials have relied on using medical visits to identify smokers and to collect the information necessary for intervention, which is resource intensive. In contrast, the current study sought to determine if cessation treatment uptake could be increased through low-intensity, mail-based outreach from the health plan, without relying on medical visits or individually-collected data to inform the intervention. Only one prior study, to our knowledge, has attempted something similar. Alesci et al. [13] randomized health plan members to receive information about access to stop-smoking medications via either their standard contract language or a direct-to-member postcard describing their coverage. One year later, there were no differences in self-reported stop-smoking medication use. These findings suggest it may take more than providing smokers with basic information about the availability of treatment to encourage its utilization. Thus, we hypothesized that combining a treatment referral with content designed to motivate smokers to consider quitting might improve subsequent treatment utilization. We also sought to address whether the intervention effects would differ by the type of motivational content or between people who receive both medical care and insurance coverage from the health plan versus those who only receive insurance coverage. While the Alesci [13] study was conducted in a mixed-model health plan which included both of these types of members, it did not look at the impact of this distinction on treatment use. However, if a difference exists, this would be important information about the generalizability of the findings to other settings, including outside the U.S. For example, findings among participants who only received insurance coverage from the health plan may be more generalizable to the general U.S. population, since most Americans do not receive health insurance coverage and healthcare from the same agency. However, findings among participants who receive both care and coverage from the same health plan may be more generalizable to settings with single-payer, universal health care coverage such as countries in the United Kingdom.

Thus, the current study explored whether enrollment in a comprehensive tobacco cessation treatment program could be improved using letters proactively mailed to smokers. Specifically, we sought to answer three questions: 1) Can proactively mailed letters encourage greater smoking cessation treatment enrollment than usual care referral strategies (i.e., no letter)?; 2) Does including motivational content in the letters make a difference compared to a letter containing referral information only?; and 3) Do the results differ between people who receive both medical care and coverage from the message source versus those who receive insurance coverage alone? The impetus for this research was the health plan’s desire to implement a mail outreach program for all smokers, but the results are relevant to others interested in promoting population health and utilization of best-practice tobacco cessation treatment services within large health care settings.

## Methods

### Setting

All activities were conducted at Kaiser Permanente Washington (KPWA; formerly, Group Health Cooperative) in Washington State. KPWA is a mixed-model health care system which provides both clinical care and insurance coverage to people in some areas of the state (i.e., Group Practice Division, GPD) and acts solely as an insurance provider to people in other state regions (i.e., network). This distinction could have implications for members’ willingness to use services, but also has implications for how smokers can be identified. In the current study, smokers in the GPD were identified via a combination of their smoking status assessed at medical appointments and ICD codes indicative of tobacco use, both available from electronic medical records (EMR). In the network, smoking status could only be identified via claims-based ICD codes indicative of recent tobacco use. All research activities took place in 2016.

### Design

The study used a pragmatic trial design, meaning the methods used reflect that of usual care and conform to the characteristics of a pragmatic trial as defined by Loudon et al. (e.g., no additional research criteria were imposed to identify smokers or screen them for eligibility, the intervention conformed to that which might be offered as a usual care practice by the health plan, no special monitoring or follow-up data collection were imposed, all data were included in the outcome analyses, and the primary outcome was directly relevant to all participants) [14]. In contrast to explanatory studies which seek to inform if an intervention can be effective when conditions are tightly controlled, pragmatic trials seek to inform intervention effectiveness under real world conditions [14, 15]. Consequently, pragmatic trials are considered more useful for informing clinical and policy decisions than highly controlled clinical trials.

### Participants

**Fig. 1:**
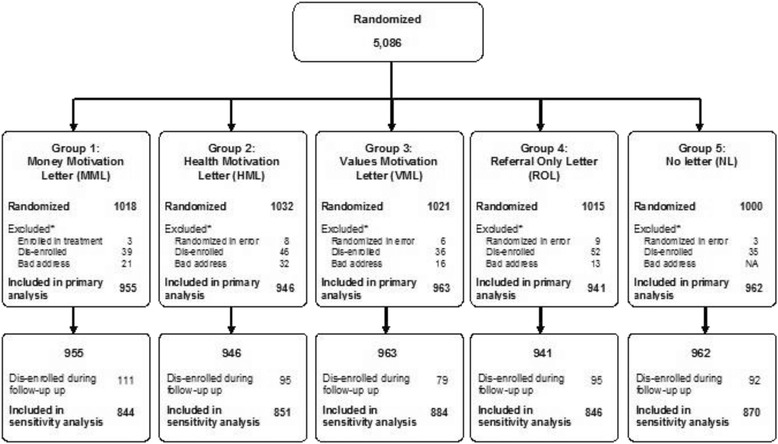
Consort diagram depicting study flow. *People may have had more than one reason for exclusion. The exclusions were applied sequentially as listed, and only the first is listed here. Enrolled in teatment incldues persons who had already enrolled in the tobacco treatment program on their letter mail date. Dis-enrolled includes people who left the health plan between the time the sample was pulled and their letter mail date. Bad address indicates the invitation letter was returned as not deliverable

Using automated data, we identified health plan members in the GPD and network who were aged 18 to 60 years old, had evidence of tobacco use based on ICD codes (all members) or a smoking flag in their electronic medical record (GPD members only), and had not enrolled in the health plan’s covered comprehensive smoking cessation program in the prior year. From this group, 5086 members were randomly selected in equal numbers across eight strata defined by sex (male vs. female), age (18–39 years old vs. 40 or older), and coverage plan (GPD vs. network). We then excluded persons with invalid mailing addresses (*n* = 82) and those who were randomized in error. The latter included persons who were already enrolled in the tobacco cessation program (*n* = 29) on their letter mail date and those who had dis-enrolled from the health plan (*n* = 208) at the time of their mail date. The final analytic sample included 4767 smokers (see Fig. 1).

All participants had access to a comprehensive tobacco cessation program which included behavioral intervention (up to 5 proactive counseling calls initiated by the quitline and unlimited future in-bound counseling calls initiated by smokers, mailed self-help materials, and access to online treatment materials) and access to pharmacotherapy (i.e., standard course of nicotine replacement therapy, varenicline, or bupropion, as medically appropriate). Access to care was a covered benefit for all.

### Randomization and intervention content

Within strata defined by age, sex, and coverage plan (see above), selected participants were randomly assigned to one of five treatment conditions in equal proportions: no letter (NL), referral only letter (ROL), health motivation letter (HML), money motivation letter (MML), or personal values motivator letter (VML). Mailed letters were chosen because email addresses were not available for all members and, as a policy, the health plan does not communicate with members via unsecure email. Letters were mailed from March to April 2016.

Each letter was of similar length (one page) and formatting, advised smokers to quit, instructed them how to enroll in the covered treatment program, was signed by a single health plan physician, and printed on health plan letterhead. Three letters included additional brief motivational content, as reflected in each letter name above. The motivational themes were chosen to reflect key reasons people quit smoking and were designed to encourage people to consider quitting based on these factors. The HML pointed out to the positive health benefits of quitting (e.g., to reduce risk of cardiovascular disease, cancer, or oral disease; prevent impotence; improve appearance, etc.) and that it is never too late to quit. The MML pointed out the amount of money one could save in one month, one year, 5 years, and 10 years if they smoke a pack a day and spend on average $8 per pack. The VML acknowledged that every person has different reasons for wanting to quit, which may include their health, finances, desire to set an example for loved ones, or other reason. Recipients were encouraged to think about their own personal reasons for wanting to quit. In accordance with Prospect Theory, which predicts that gain-framed messages will be more influential for changing preventative health behaviors than are loss-framed messages, the motivational letters were written in a gain-framed tone (e.g., quitting smoking can help you stay strong and vibrant, prevent disease, or save money) [16]. In contrast, the ROL instructed people how to enroll in the covered treatment program, but did not include gain-framed or motivational content.

### Assessment and analyses

Enrollment in the covered tobacco cessation program was monitored for 5 months following the letter mail date for each person. Letters were mailed in weekly waves. Each wave included participants from each arm to ensure equitable distribution across time. Persons in the NL letter arm were similarly assigned to weekly mail waves in order to establish their index “mail date,” although no letters were mailed to this arm. Treatment utilization was determined via automated enrollment records. Given the small cell sizes, group differences were compared using Fisher’s exact test. Risk differences (differences in the proportion enrolling between groups) and confidence intervals were also calculated to estimate the effect size of each letter condition. As a sensitivity analysis, group comparisons were repeated after excluding participants who dis-enrolled from the health plan during the 5-month follow-up period (*n* = 472), since they did not have the same access to treatment as others.

## Results

Among participants randomized to one of the four letter conditions (all groups combined), 0.8% of participants enrolled in the tobacco treatment program during the observation period compared to 0.9% in the NL group (*p* = .69). Enrollment across the different letter conditions ranged from 0.5% in the MML group to a 1.1% in the ROL group (Table 1). Differences across arms were not statistically significant (*p* = .71) and all effect sizes were small.

**Table 1:** Treatment Enrollment by Randomization Group

	Enrolled in Treatment within 5 months^a^								
	All participants			Network participants^b^			GPD participants^b^		
	N	n (%)	Risk Difference (95% CI)^c^	N	n (%)	Risk Difference (95% CI)^c^	N	n (%)	Risk Difference (95% CI)^c^
No letter (NL)	962	9 (0.9)	Referent	422	1 (0.2)	Referent	540	8 (1.5)	Referent
Money (MML)	955	5 (0.5)	− 0.004 (− 0.012, 0.003)	410	1 (0.2)	0.000 (− 0.007, 0.007)	545	4 (0.7)	− 0.007 (− 0.020, 0.005)
Health (HML)	946	7 (0.7)	− 0.002 (− 0.010, 0.006)	441	1 (0.2)	− 0.000 (− .006, 0.006)	505	6 (1.2)	− 0.003 (− 0.017, 0.011)
Values (VML)	963	9 (0.9)	0.000 (− 0.009, 0.009)	425	3 (0.7)	0.005 (− 0.005, 0.014)	538	6 (1.1)	− 0.004 (− 0.017, 0.010)
Referral (ROL)	941	10 (1.1)	0.001 (− 0.008, 0.010)	418	1 (0.2)	0.000 (− 0.007, 0.007)	523	9 (1.7)	− 0.002 (− 0.013, 0.017)

^a^Enrollment in comprehensive tobacco treatment program within 5 months post-letter (*n* = 4767)

^b^Group Practice Division (GPD) participants received care and coverage from the health plan. Network participants received insurance coverage only from the health plan

^c^*CI* Confidence interval

Overall, GPD participants were more likely to enroll in treatment than network participants (1.2% vs. 0.3%, *p* < .01), but no specific letter had a statistically significant advantage among GPD or network subgroups. Exclusion of participants who dis-enrolled from the health plan during the observation period did not alter the findings (data not shown).

## Discussion

Increasing uptake of evidence-based smoking cessation treatment is an important public health goal. Health care systems and insurance providers are particularly well-positioned to support this goal given their ability to identify smokers using automated data and connect them with covered treatment services.

We did not find that proactively mailing smokers a one-time letter increased treatment uptake compared to usual care (i.e., not receiving a letter). Moreover, no specific letter content (whether motivational or referral only) was superior to the others. Not only were the differences not statistically significant, no comparisons differed by more than 1%, which calls into question whether any of the letters would be cost-effective to implement as a “real-world” intervention. At best, the letters would result in an additional 10 treatment seekers (maximum) per 1000 likely smokers contacted.

The current trial was motivated by the health plan’s intent to implement a similar mail outreach program as usual care. These plans were dropped in light of the study findings, but the results have relevance to other health care systems, health insurance providers, policy makers, and researchers interested in promoting utilization of evidence-based treatment among smokers using low-cost, population-level interventions. Alesci et al. [13] also failed to find that a postcard notification increased self-reported use of stop smoking medications among health plan members. Taken with the results of the current study, these findings suggest that minimal-intensity, proactive mailers--even those that include motivational content and referral information for covered treatments--are not likely to meaningfully increase smoking cessation treatment uptake. The comparison among our two subsamples (GPD vs. network) offers some additional insight into the generalizability of these findings to other settings. Treatment enrollment was significantly higher among smokers in the GPD than those in the network. This suggests proactively-mailed referral letters may be more effective when the referral comes from a single-agency that provides both care and coverage, such as integrated health care systems in the U.S. or single-payer, universal health care providers in other countries. However, given the low overall rate of enrollment in the GDP (1.2% of smokers contacted), it is still difficult to advocate for adoption of this single contact, minimal intervention strategy in these settings.

The findings also speak to a fundamental challenge--most smokers are ambivalent about quitting smoking. That is, they want to quit smoking someday, but are not yet ready to quit or ready to seek treatment [6]. Given this and the fluid nature of motivation, which can change from moment to moment based on one’s situational context [17], minimal-intensity interventions may be more effective if they involve repeated contacts over time. If these contacts use electronic communications like SMS text messaging or emails, this could increase the cost-effectiveness of this intervention strategy. However, electronic communication requires access to smokers’ mobile phone numbers or email addresses and use of these communication channels may be restricted by applicable privacy laws such as the U.S. Health Insurance Portability and Accountability Act, which limits what personal health information certain agencies (like health providers and insurers) can share with people using unsecured electronic communications. As such, this may not be a viable strategy in all settings.

This study has several strengths including its real-world setting and pragmatic design; evaluation of a standardized, low-intensity intervention which could be replicated by others with access to population-level data on smokers; use of automated data to identify participants and confirm treatment utilization; and a sound methodological design. A limitation was that we only assessed use of the comprehensive tobacco treatment program available to health plan members and to which everyone in a letter intervention arm was referred. Best practice dictates that smokers be offered comprehensive treatment which includes counseling and pharmacotherapy, [5] but it is possible that some participants who received a letter sought pharmacotherapy without enrolling in the provided treatment program. If so, our results would under represent the motivational impact of the letters on behavior change. However, since smokers can purchase over-the-counter stop smoking medications out of pocket without using their insurance coverage, it is impossible to accurately assess the effects of the intervention on medication use in this pragmatic study since it did not include self-reported data collection. Future studies should consider alternative strategies to account for this.

## Conclusions

In sum, increasing the use of evidence-based, comprehensive tobacco treatment programs is an important public health goal. While large health care systems have the resources and infrastructure to identify smokers and proactively encourage treatment utilization, minimal mail-based interventions are not an effective or cost-effective strategy for achieving this goal. Future research should explore whether low-cost electronic communications or alternative proactive outreach strategies may be more effective.

## Abbreviations

GPD: Group practice division
HML: Health motivation letter
ICD: International Statistical Classification of Diseases and Related Health Problems
KPWA: Kaiser Permanente Washington
MML: Money motivation letter
NL: No letter
ROL: Referral only letter
VML: Personal values motivation letter
